# Comparison of consumer-grade wearable devices with a research-grade instrument for measuring physical activity in a free-living setting

**DOI:** 10.1371/journal.pone.0342543

**Published:** 2026-02-23

**Authors:** Takuya Miwa, Kazuma Mii, Ryouichi Chatani, Yasuo Sugitani

**Affiliations:** 1 Translational Research Division, Chugai Pharmaceutical Co., Ltd., Tokyo, Japan; 2 Clinical Development Division, Chugai Pharmaceutical Co., Ltd., Tokyo, Japan; Japanese Academy of Health and Practice, JAPAN

## Abstract

**Introduction:**

Wearable accelerometer devices are now widely used in both research and daily life settings. This study aimed to compare the accuracy of three commercially available consumer-grade activity monitors with the medical-grade ActiGraph device in a free-living setting in Japan.

**Methods:**

Thirty-six office workers were enrolled and provided with an ActiGraph. Data were analyzed from participants who also wore Apple Watch (*n* = 21), Fitbit (*n* = 22), and Oura Ring (*n* = 5) over a 3-week period. Step count, physical activity energy expenditure (PAEE), and moderate-to-vigorous physical activity (MVPA) data were collected from all devices. Data were analyzed using correlation coefficients, mean differences, and Bland–Altman plots.

**Results:**

ActiGraph data confirmed comparable physical activity levels across the participant subgroups, ensuring a valid basis for the subsequent inter-device comparisons. Step counts were largely consistent across devices, with Apple Watch and Oura Ring measurements within 10% of ActiGraph measurements (mean percentage differences 2.12% and −6.24%, respectively), while the Fitbit overestimated step count by 18.00%. MVPA showed greater variability, with Apple Watch and Oura Ring underestimating by 46.22% and 11.64% respectively, whereas the Fitbit showed minimal mean difference (0.62%). PAEE showed the largest discrepancies, with Apple Watch and Fitbit overestimating by 25.91% and 139.19% respectively, and Oura Ring underestimating by 16.87%. Correlation coefficients were strong for step counts (*r* = 0.84–0.92) but lower for MVPA and PAEE across all devices. Bland–Altman analysis revealed proportional bias in the Fitbit’s PAEE and the Apple Watch’s MVPA, with errors increasing at higher activity levels.

**Conclusion:**

Step counts were largely consistent with the ActiGraph for most devices; however, the Fitbit showed a notable overestimation. However, the ability of those devices to accurately measure MVPA and PAEE appeared to be more limited, particularly at higher activity levels. These findings underscore that the selection of a consumer-grade wearable for research or clinical use must be carefully guided by the specific metric of interest. However, the findings for the Oura Ring should be interpreted with caution due to the small sample size.

## Introduction

Several wearable accelerometer devices designed to quantify physical activity are currently available and are widely used by consumers in the daily life as well as in research settings [1]. Some wearable devices, such as the ActiGraph activity monitors and the Empatica E4 wristband, are validated medical-grade devices and have been used in multiple clinical trials. For example, ActiGraph products have been in use for more than two decades and have been featured in more than 20,000 research articles [2]. However, applying these research-grade instruments in daily life studies has some limitations, including that they are often large and expensive.

Outside of the research setting, there are a growing number of consumer-grade wearable devices available that can be used in combination with smartphone and computer applications to monitor and manage personal health [3]. These devices are now being used routinely in daily life studies to monitor movement and exercise in daily life [1]. However, data obtained from various devices may not be equivalent because of differences in the device sensors and in the algorithms used to calculate measures of activity. Moreover, factors such as wearing position (wrist vs hip vs ankle), physical characteristics of the user, and the ability to customize the device to calibrate for specific activities also affect accuracy [4]. Furthermore, although several studies have investigated inter-device accuracy and reliability, most have only monitored participants over several days or a week [5–9]. However, in clinical trials and daily life settings, such short periods are often insufficient to accurately assess disease progression, treatment effects, and behavioral changes over time, and longer monitoring is crucial. Extended observation periods provide more clinically relevant insights into patient activity patterns, enhancing the utility of wearable devices in clinical research and practice.

While the ActiGraph GT3X has historically served as a common comparator for evaluating consumer-grade devices [5–7,9,10,11], the research field is transitioning to the newer ActiGraph GT9X. The GT9X is a more advanced device, equipped with additional sensors such as a gyroscope and magnetometer [12]. Importantly, its accuracy depends on wear location: studies have shown that the GT9X provides reliable step counts when worn at the hip or ankle, but is less accurate at the wrist [13]. Despite this shift, data directly comparing the GT9X against popular consumer wearables in free-living settings remain limited [14].

This study was therefore designed to address several specific gaps in the current literature. First, it provides a direct, multi-device comparison against the newer ActiGraph GT9X, adding to the limited body of evidence for this device [14]. Second, whereas most validation studies are conducted over short periods [5–9], our study utilizes a longer 3-week free-living period to better capture habitual activity patterns. Third, it addresses the scarcity of such validation studies conducted specifically within a Japanese population, focusing on the relevant demographic of office workers. Finally, we include the Oura Ring, a popular device that has been less frequently validated against a research-grade standard compared to the Apple Watch and Fitbit. The objective of this study was thus to address these gaps by evaluating the quality and reliability of data from the Apple Watch, Fitbit Sense, and Oura Ring in comparison to the ActiGraph GT9X among Japanese office workers.

## Materials and methods

### Participants

This study recruited volunteers who were employees (office workers) of the study sponsor (Chugai Pharmaceutical Co., Ltd.) from May 18–19, 2021. The recruitment period was closed after two days as the target number of participants was quickly reached. No monetary or other material compensation was provided for participation. Eligible participants had to consent to participate in the study, regularly engage in exercise at least once per week as part of their usual routine, and be willing to wear physical activity tracking devices throughout the study period. For this study, exercise was defined according to the guidance of the Japanese Ministry of Health, Labour and Welfare as any physical activity (excluding swimming) that was performed in a planned and intentional manner to maintain or improve physical fitness [15]. Individuals with a history of rashes or other problems related to the wearing of a device were ineligible to participate in the study. In addition, individuals who were involved in the study planning, execution, or subsequent analyses were also ineligible to participate. No other exclusion criteria, including those related to health conditions, were applied to the participants.

### Ethical considerations

Ethical approval was granted by the Ethics Committee of Chugai Pharmaceutical (registered with the Ministry of Health, Labour and Welfare, Japan [Registration Number 11001059]; Approval Number, E21004) and all participants provided written informed consent prior to participation. The study complied with the Ethical Guidelines for Medical and Health Research Involving Human Subjects and the Act on the Protection of Personal Information [16,17]. All data collected from participants were anonymized so that individuals could not be identified.

To ensure voluntary participation and prevent potential coercion, the study team was functionally separated. A designated study coordinator, who was not involved in data analysis or interpretation, handled all direct interaction with participants, including recruitment and the consent process. The researchers (authors) were blinded to participant identities and only had access to anonymized data. It was also explicitly stated during the consent process that participation was voluntary and that the decision to participate, or to withdraw from the study at any time, would not result in any disadvantageous treatment.

### Wearable devices

The devices that we evaluated in this study were Apple Watch Series 6 (Apple Inc., Cupertino, CA, USA), Fitbit Sense (Fitbit Inc., San Francisco, CA, USA), Oura Ring 3 (Oura Health Oy, Oulu, Finland), and ActiGraph GT9X (ActiGraph LLC., Pensacola, FL, USA). Features of each device are summarized in Table 1. All wrist-worn devices were worn at the same time on the wrist of the non-dominant hand for the duration of the study. The ActiGraph was worn on each participant’s hip during the day and on their dominant wrist while sleeping, per the device specifications for collecting sleep-related actigraphy data. The Oura Ring could be worn on any finger. Participants were asked to wear their devices for as much time as possible except when charging the device or bathing.

**Table 1:** Summary of features of the four fitness tracking devices used in this study.

Device	Manufacturer	Wearing position	Biomarkers/ vital signs measured	Physical activity measured
Apple Watch Series 6	Apple Inc., Cupertino, CA, USA	Wrist^*a*^	• Blood oxygen levels • Heart rate • Electrocardiogram	• Step count • Sleep time • TDEE • PAEE • Exercise minutes
Fitbit Sense	Fitbit Inc., San Francisco, CA, USA	Wrist^*a*^	• Skin temperature • Skin potential • Heart rate	• Step count • Sleep time • TDEE • MVPA
Oura Ring 3	Oura Health Oy, Oulu, Finland	Finger	• Body temperature • Heart rate • Respiratory rate	• Step count • Sleep cycles • TDEE • PAEE • MVPA
ActiGraph GT9X	ActiGraph LLC., Pensacola, FL, USA	Hip^*a*^	• — ^*c*^	• Step count • Sleep time • TDEE • PAEE • MVPA

^a^Worn on the non-dominant hand.

^b^Worn on the dominant hand while sleeping.

^c^Heart rate can be measured via an additional attachment. Heart rate was not monitored in this study.

Abbreviations: MVPA, moderate-to-vigorous physical activity; PAEE, physical activity energy expenditure; TDEE, total daily energy expenditure.

### Study design

The focus of this study was to compare physical activity data between consumer-grade devices and the ActiGraph. All 36 participants wore the ActiGraph GT9X device. Of these 36 participants, 12 were chosen at random to also wear the Fitbit Sense, 12 to wear the Apple Watch, and 12 to wear both the Apple Watch and the Fitbit. A subgroup of participants also wore the Oura Ring; these were selected based on having appropriately sized fingers for the limited number of Oura Rings available.

The study was conducted at Chugai Pharmaceutical Co., Ltd., Japan between June 2021 and September 2021. A total of 36 participants were recruited and randomly allocated on Day −7. After randomization, participants completed a 1-week run-in period (Days −7 to −1) to become familiar with wearing the devices and using the associated smartphone applications. During this time, the participants wore their assigned devices and it was confirmed that the devices were connected and operating correctly, but no data were analyzed.

Following the run-in period, the study proceeded with a 21-day observation period (Days 1–21). During this time, data were collected from all devices for analysis. Participants were instructed to wear their assigned devices continuously, except when bathing or when charging was necessary. They were asked to maintain their normal daily activities and sleep patterns throughout the study period.

### Outcomes and statistical analyses

During the study, data on the participants’ physical activity, sleep, and vital signs were collected from each device. However, only data on physical activity (step count, physical activity energy expenditure [PAEE], and moderate-to-vigorous physical activity [MVPA]) were evaluated in the analyses presented here. Data from the consumer devices were obtained through their standard data access methods. Specifically, Apple Watch data were exported as XML files directly from the Apple Health application. Fitbit data were retrieved as minute-level time-series data via the official Fitbit Web API, and Oura Ring data were similarly accessed via the Oura Cloud API. Data for the criterion ActiGraph device were downloaded using the CenterPoint cloud platform. As the granularity and structure of the raw data differed between devices (see S1 Table), all datasets were processed and aggregated into daily summary values for step count, MVPA, and PAEE to allow for direct comparison. Due to differences in data output across devices, some measurements required substitution or conversion for comparison. As the Apple Watch does not provide a direct MVPA output based on standard research definitions, we used its “Exercise minutes” metric—defined by the manufacturer as activity equivalent to or exceeding a brisk walk—as a pragmatic proxy to facilitate a comparison with the MVPA data from the other devices. It should be noted that these metrics are not considered directly equivalent. Likewise, the Fitbit does not record PAEE; therefore, PAEE was calculated by subtracting each participant’s basal metabolic expenditure from their total daily energy expenditure (TDEE), following the method described in a previous study [18]. Wear time was determined using the ActiGraph data as the reference for all concurrently worn devices. The mean daily wear times were comparable across all device types (see S2 Table for details).

Data were analyzed for three groups based on which commercial wearable device the participants wore (Apple Watch, Fitbit, or Oura Ring). Participant demographics and baseline characteristics (age, sex, body weight, height, body mass index [BMI], concomitant medication use, dominant hand, and presence of comorbidities) were summarized for each group. To compare devices, data obtained from each device were summarized as daily means or medians. In addition, scatter plots and Bland–Altman plots were created with all daily data.

To evaluate longer-term performance, the mean and median number of steps, MVPA, and PAEE per day were calculated for each commercial device over a 3-week period. Spearman’s correlation coefficient, Pearson’s correlation coefficient, mean difference, median difference, mean percentage difference, and median percentage difference for devices were calculated for each comparison. For all analyses, daily data were included only if the ActiGraph wear time was at least 10 hours. This single criterion was applied to data from all devices under the assumption of concurrent wear, as participants were instructed to wear all devices simultaneously and the consumer-grade devices do not provide accessible wear time metrics for validation. Days with zero values recorded for the consumer devices were also excluded.

The sample size was determined based on feasibility rather than a formal statistical hypothesis. All statistical analyses were performed using Python (version 3.10.10) and relevant libraries including NumPy, pandas, and SciPy. Formal normality testing of the data was not performed. This approach was chosen because physical activity sensor data are typically non-normally distributed and the primary study objective was descriptive agreement analysis rather than formal hypothesis testing. Therefore, to provide a comprehensive view, both mean and median were reported for central tendency. Similarly, both Spearman’s and Pearson’s correlation coefficients were presented; Spearman’s correlation was considered the primary measure of monotonic agreement, while Pearson’s correlation was included as supplementary information to assess the degree of linear relationship.

## Results

### Participants

**Fig 1:**
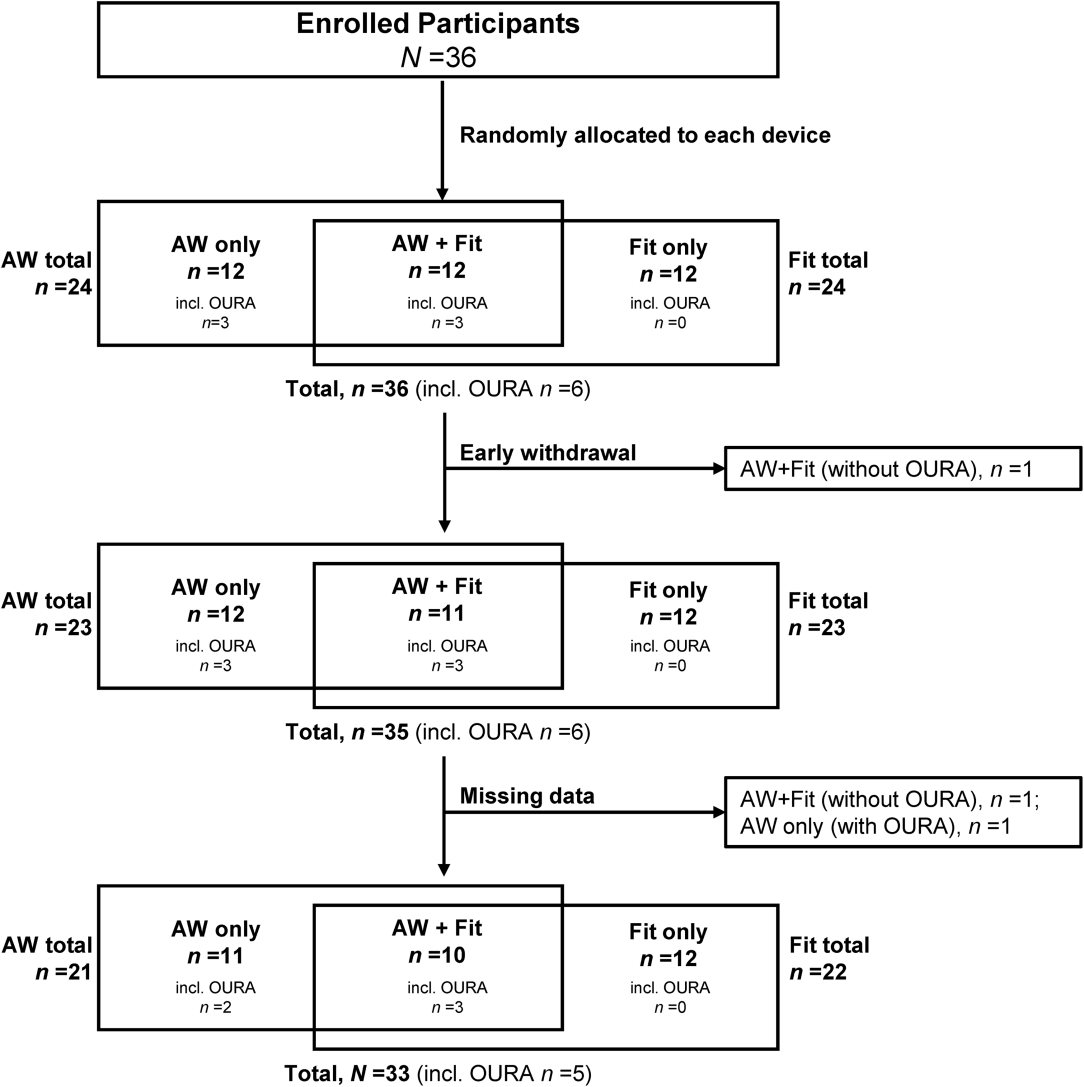
Participant disposition. Abbreviations: AW, Apple Watch; Fit, Fitbit; OURA, Oura Ring.

The study enrolled 36 participants who were randomly allocated each device. One participant allocated both an Apple Watch and a Fitbit withdrew from the study early because of difficulties in wearing the ActiGraph. Two participants (one patient allocated both an Apple Watch and a Fitbit and the other allocated only an Apple Watch) had missing data owing to <10 hours of wearing or device malfunction. In total, 21 participants provided data for the Apple Watch and ActiGraph (10 participants were allocated both the Apple Watch and Fitbit and 11 participants were allocated only the Apple Watch), 22 participants provided data for the Fitbit and ActiGraph (10 were allocated both the Apple Watch and Fitbit and 12 were allocated only the Fitbit), and five participants provided data for the Oura Ring and ActiGraph (three of these five participants were allocated both the Apple Watch and Fitbit and two were allocated only the Apple Watch) (Fig 1).

Across the overall study population (*N* = 33), participants (21 men, 12 women) had a mean age of 41.79 years (range: 27–59), had a normal BMI (mean 23.04 kg/m^2^), and 33.30% reported comorbidities(e.g., well-controlled hypertension or dyslipidemia), which were of a non-serious nature and deemed unlikely to affect the results as the study’s inclusion criteria required all participants to be physically active (Table 2). Baseline characteristics were broadly comparable among participants wearing the Apple Watch, Fitbit, or Oura Ring devices.

**Table 2:** Baseline demographics and characteristics of the participants, overall and by use of consumer-grade wearable activity monitoring device.

	Total (*N* = 33)	Apple Watch (*n* = 21)	Fitbit (*n* = 22)	Oura Ring (*n* = 5)
Age, years	41.79 ± 8.05 (27-59)	41.10 ± 8.48 (27-59)	43.41 ± 7.5 (27-56)	47.40 ± 5.03 (41-53)
Sex, male	21 (63.64)	14 (66.67)	13 (59.09)	3 (60.00)
Body weight, kg	65.15 ± 12.32	64.76 ± 11.86	66.45 ± 13.76	58.60 ± 9.61
Height, cm	167.85 ± 7.59	168.62 ± 8.14	166.50 ± 8.17	166.80 ± 9.18
Body mass index, kg/m^2^	23.04 ± 3.63	22.69 ± 3.33	23.82 ± 3.87	21.02 ± 2.64
Concomitant medication use, yes	11 (33.33)	6 (28.57)	7 (31.82)	1 (20.00)
Dominant hand, right	31 (93.94)	21 (100.00)	20 (90.91)	5 (100.00)
Presence of comorbidities, yes	10 (30.30)	6 (28.57)	7 (31.82)	2 (40.00)

Data are presented as mean ± standard deviation or *n* (%); for age, data are presented as mean ± standard deviation (range).

### ActiGraph data

Physical activity data collected by the ActiGraph were analyzed in subgroups defined by which consumer-grade device the participants wore (Table 3). For each subgroup (Apple Watch vs Fitbit vs Oura Ring), mean values were similar with respect to the amount of time the ActiGraph was worn (17.58 vs 18.10 vs 17.53 hours/day), step count (7610.77 vs 7516.32 vs 8007.73 steps/day), MVPA (52.39 vs 50.92 vs 55.93 minutes/day), and PAEE (368.27 vs 367.34 vs 380.45 kcal/day), and, indicating good adherence to wearing the device and similar levels of physical activity among the subgroups.

**Table 3:** Summary statistics for the activity data from consumer-grade wearable devices vs ActiGraph data.

Output	Commercial device (no. of data values^*^)	ActiGraph data		Commercial device data		Comparison (ActiGraph vs commercial device)					
		Mean (SD)	Median	Mean (SD)	Median	Spearman’s rank correlation coefficient	Pearson’s correlation coefficient	Mean difference	Median difference	Mean difference (%)	Median difference (%)
Step count (steps/day)	Apple Watch (*n* = 371)	7625.67 (4815.90)	6987.00	7787.53 (4833.09)	7063.00	0.84	0.86	161.86	76.00	2.12	1.09
	Fitbit (*n* = 364)	7512.99 (5270.77)	6396.00	8864.96 (5909.51)	7737.00	0.92	0.92	1351.97	1341.00	18.00	20.97
	Oura Ring (*n* = 69)	7814.06 (3783.65)	7466.00	7326.22 (3580.10)	7267.00	0.92	0.91	−487.84	−199.00	−6.24	−2.67
MVPA (minutes/day)	Apple Watch (*n* = 348)	53.32 (44.21)	44.00	28.68 (26.05)	22.50	0.68	0.57	−24.64	−21.50	−46.22	−48.86
	Fitbit (*n* = 262)	62.60 (46.48)	54.50	62.98 (60.88)	47.50	0.70	0.79	0.39	−7.00	0.62	−12.84
	Oura Ring (*n* = 66)	57.27 (35.64)	46.50	50.61 (30.23)	47.00	0.82	0.78	−6.67	0.50	−11.64	1.08
PAEE (kcal/day)	Apple Watch (*n* = 371)	365.87 (247.36)	306.22	460.68 (187.99)	420.15	0.64	0.54	94.81	113.93	25.91	37.21
	Fitbit (*n* = 364)	367.38 (244.84)	311.76	878.75 (520.09)	796.31	0.60	0.71	511.37	484.55	139.19	155.42
	Oura Ring (*n* = 69)	359.55 (153.29)	322.85	298.90 (152.37)	292.00	0.64	0.56	−60.65	−30.85	−16.87	−9.56

Devices used include the ActiGraph GT9X, Apple Watch Series 6, Fitbit Sense, and Oura Ring 3.

* Data value represents valid utilization data per participant per day. The total count of valid daily data units is aggregated for each device.

MVPA, moderate-to-vigorous physical activity; PAEE, physical activity energy expenditure; SD, standard deviation.

### Comparisons between data from ActiGraph and consumer-grade wearable devices

**Fig 2:**
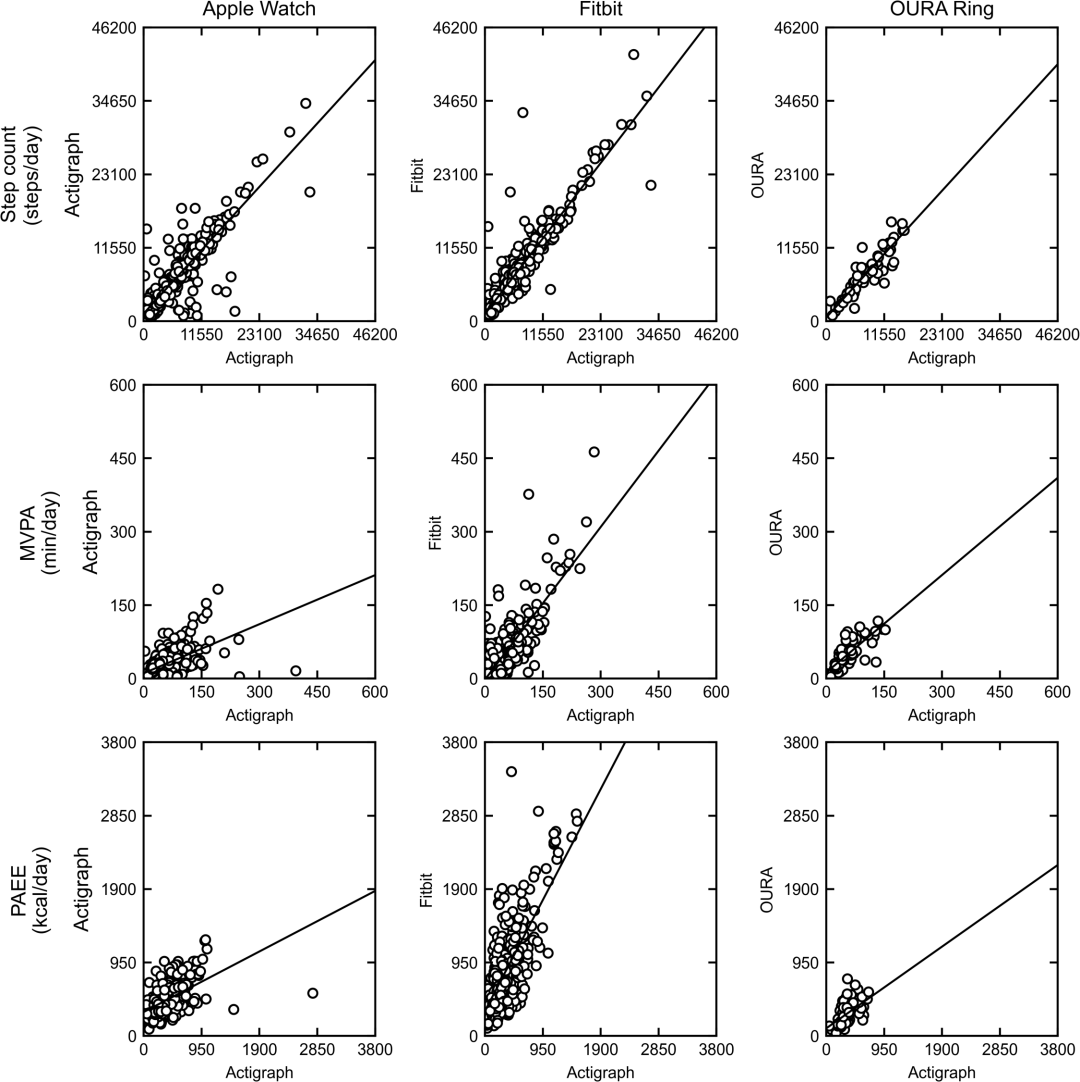
Scatter plots comparing activity data from the ActiGraph GT9X device with those of consumer-grade wearable devices. The panels are arranged by metric in rows (top: step count; middle: MVPA; bottom: PAEE) and by device in columns (left: Apple Watch; middle: Fitbit; right: Oura Ring). Each panel displays a scatter plot comparing data from a consumer-grade device (y-axis) against the criterion ActiGraph GT9X (x-axis). The solid line in each plot represents the line of best fit from linear regression.

Step counts, MVPA, and PAEE data for each of the three commercial devices and a comparison of the ActiGraph vs consumer-grade device data are shown in Table 3, and as scatter plots in Fig 2.

The correlations between the consumer devices and ActiGraph varied depending on the physical activity metric (Table 3). All devices showed strong correlations with ActiGraph for step counts (Spearman’s *ρ* = 0.84–0.92; Pearson’s *r* = 0.86–0.92). In contrast, the correlations were generally lower for MVPA (*ρ* = 0.68–0.82; *r* = 0.57–0.79) and PAEE (*ρ* = 0.60–0.64; *r* = 0.54–0.71). This indicates that the agreement between the devices was stronger for a simple metric like step counts than for more complex metrics like MVPA and PAEE.

For step count, the Apple Watch and Oura Ring matched the ActiGraph closely, with the Apple device only slightly overestimating the mean step count (7787.53 vs 7625.67 with the ActiGraph; mean percentage difference, 2.12%). In the small Oura Ring subgroup (*n* = 5), the data suggested a slight underestimation (7326.22 vs 7814.06; mean percentage difference, −6.24%). The Fitbit overestimated the step count vs the ActiGraph (8864.96 vs 7512.99; mean percentage difference, 18.00%).

The Apple Watch substantially underestimated MVPA compared with the ActiGraph (28.68 vs 53.32 minutes/day; mean percentage difference, −46.22%). In contrast, the mean MVPA measured by Fitbit showed excellent agreement with the ActiGraph (62.98 vs 62.60 minutes/day; mean percentage difference, 0.62%), while in the Oura Ring subgroup (*n* = 5), it was slightly underestimated by the Oura Ring (50.61 vs 57.27; mean percentage difference, −11.64%).

The Apple Watch overestimated PAEE with a mean percentage difference of 25.91% (460.68 vs 365.87 kcal/day). In the small cohort of Oura Ring users (*n* = 5), the device appeared to underestimate PAEE to a similar degree (298.90 vs 359.55 kcal/day; mean percentage difference, −16.87%). A considerable overestimate was noted with the Fitbit (878.75 vs 367.38 kcal/day; mean percentage difference, 139.19%).

**Fig 3:**
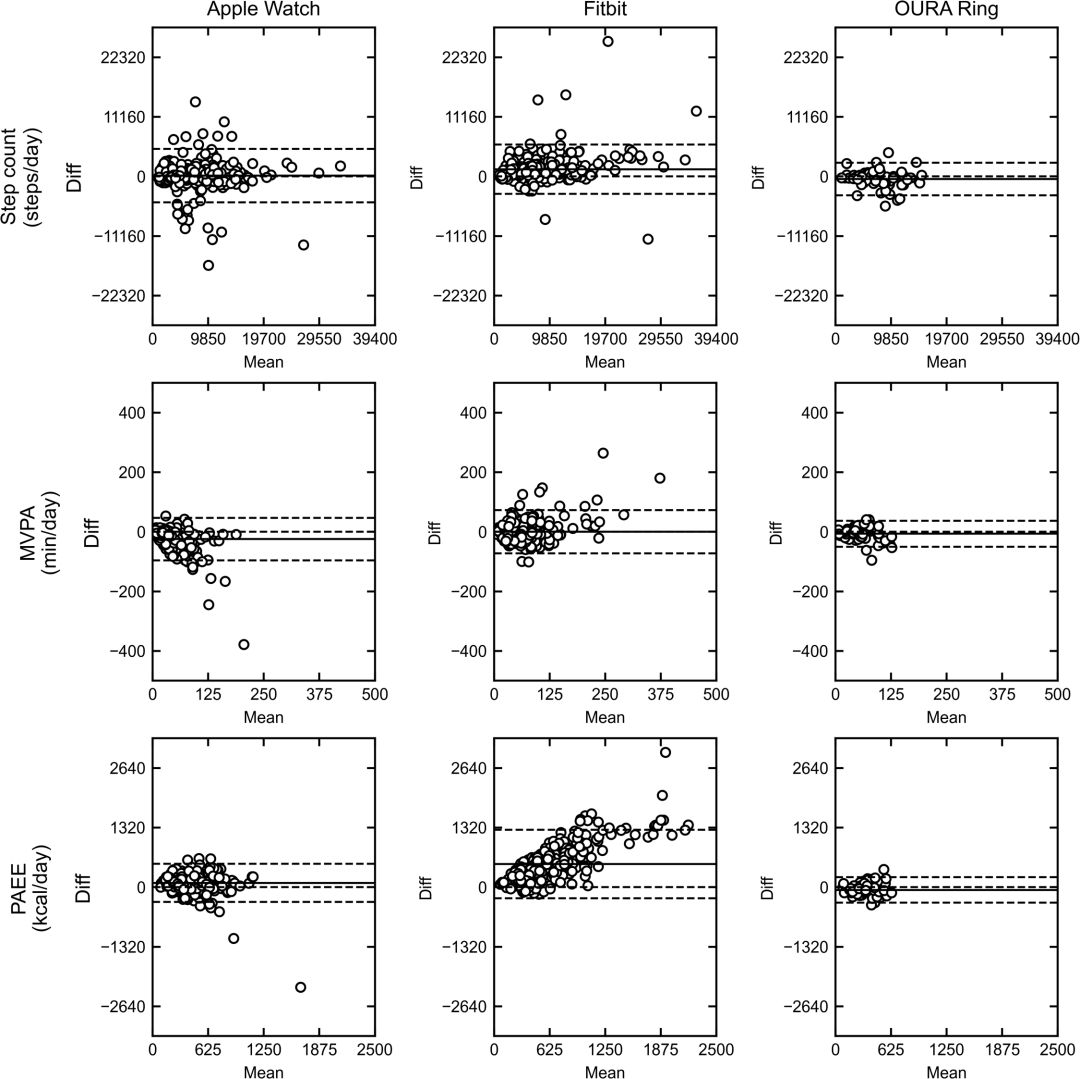
Bland–Altman plots comparing activity data from the ActiGraph GT9X device with those of consumer-grade wearable devices. The panels are arranged by metric in rows (top: step count; middle: MVPA; bottom: PAEE) and by device in columns (left: Apple Watch; middle: Fitbit; right: Oura Ring). In each plot, the y-axis shows the difference between the two devices (consumer device − ActiGraph), and the x-axis shows the mean of their measurements ([consumer device + ActiGraph]/2). The three horizontal dashed lines represent the mean difference (bias; central line) and the 95% LoA (upper and lower lines), calculated as the mean difference ± 1.96 SD of the differences.

The Bland–Altman (BA) analysis revealed varying levels of agreement for each metric (Fig 3). For step counts, no significant proportional biases were observed. The Limits of Agreement (LoAs) for Apple Watch and Fitbit were comparable and wider than those of the small Oura Ring subgroup (*n* = 5). For MVPA, proportional biases were evident for two devices. The Apple Watch showed a trend of increasing underestimation as the mean MVPA increased. Conversely, Fitbit displayed a slight trend of increasing overestimation with higher mean values. The Oura Ring subgroup, which requires cautious interpretation, had narrower LoAs and fewer outliers than the other devices. For PAEE, Fitbit demonstrated a clear proportional bias (*r* = 0.45, *p* < 0.001), indicating that the device progressively overestimated energy expenditure as activity levels rose. This finding was accompanied by a substantial overestimation bias and the widest LoAs among the devices. In contrast, Apple Watch and Oura Ring did not show significant proportional bias, with most of their data points falling within their respective LoAs. This proportional bias is particularly relevant for studies involving highly active individuals, as it indicates that Fitbit may substantially misestimate energy expenditure at higher activity levels.

## Discussion

This study aimed to compare the accuracy of three popular consumer-grade wearable devices (Apple Watch, Fitbit Sense, and Oura Ring) with the research-grade ActiGraph GT9X for measuring physical activity in a free-living setting among Japanese office workers. We evaluated step count, MVPA, and PAEE over a 3-week period. Thirty-six participants wore the ActiGraph GT9X along with one or more consumer devices, engaging in their normal daily activities including at least one exercise session per day. Our analysis revealed that while step counts were largely consistent between the ActiGraph and consumer devices, there were notable discrepancies in MVPA and PAEE measurements. These findings highlight the importance of understanding device-specific characteristics when selecting wearables for research or clinical applications.

In this study, the ActiGraph GT9X was employed as the criterion measure to evaluate the performance of consumer wearable devices. The ActiGraph series of accelerometers is widely recognized as a reference device in physical activity research, and its validity and reliability for measuring step counts, MVPA, and PAEE have been extensively documented in numerous studies under both laboratory and free-living conditions [2,12,19]. Therefore, we selected the ActiGraph GT9X to provide a robust and scientifically accepted benchmark against which the consumer devices could be compared.

To date, we have identified few published studies that compared consumer-grade wearable devices (Fitbit Charge 2 and Garmin vivosmart HR+) with the ActiGraph GT9X in a free-living environment [14]. Although that study was performed in a population of older adults (aged ≥65 years), activity levels as defined by step count measured by the ActiGraph GT9X were comparable with those of our study (8375.70 vs 7512.99–7814.06 steps/day) [14], and all devices were highly consistent in terms of step count, which is also consistent with our results. Compared with the reference tracker (New-Lifestyles NL-2000i tracker), the Fitbit Charge 2 tended to overestimate the step count (mean percentage error: 12.36%), which again is consistent with our findings for the Fitbit Sense (mean difference: 18.00%). In both studies, Fitbit devices overestimated MVPA compared with the ActiGraph GT9X. Our study also has the benefit of analyzing data generated continuously over 3 weeks. In contrast, other studies using the ActiGraph GT3X have used a much shorter duration, e.g., 24 hours [5,6], 2 days [7,8], or 7 days [9,10,11].

In this study, step count was the parameter that showed the highest consistency between the ActiGraph and consumer-grade devices among all activity parameters measured. The Apple Watch and Oura Ring provided step count readings within 10% of those determined by the ActiGraph, while the Fitbit tended to overestimate. Regarding MVPA, the Apple Watch substantially underestimated this parameter compared to the ActiGraph. The performance of all three commercial devices showed the largest discrepancies for PAEE measurements, with the Apple Watch and Fitbit overestimating by 25.91% and 139.19%, respectively, and the Oura Ring underestimating by 16.87%. These findings highlight significant differences between devices in measuring MVPA and PAEE, while step count evaluations yielded relatively consistent results across different devices.

The visualization of error trends using Bland–Altman plots revealed accuracy tendencies dependent on the measurement range. The most notable was the PAEE measurements with the Fitbit, which showed clear proportional bias. In contrast, step count and MVPA measurements by the same device did not show such pronounced trends. Although less prominent than the Fitbit’s PAEE, the Apple Watch’s MVPA measurement also exhibited proportional bias. It is worth noting that for the Apple Watch, which does not directly output MVPA, we used “Exercise minutes” as a proxy. Similarly, for the Fitbit’s PAEE, we followed the approach of previous studies [18] by subtracting basal metabolic expenditure from the total daily energy expenditure (TDEE) output. These methodological adaptations may have influenced the observed errors. These findings underscore the importance of considering device-specific tendencies when using these wearables in daily life applications or clinical settings. The accuracy and reliability of measurements can vary significantly depending on the specific parameter being measured and the device being used.

A key methodological consideration of this study is the difference in wear location between the criterion ActiGraph (hip) and the consumer devices (wrist/finger). While measurements can differ between the hip and wrist, our approach is consistent with the standard protocol used in numerous validation studies comparing consumer wearables to a research-grade device [5–7,9,10]. This choice was also practical; requiring participants to wear an additional device on the wrist would have increased participant burden and could have influenced their natural movement patterns. Indeed, this difference in wear location is a likely contributor to the discrepancies observed, providing important context for the findings discussed below.

Several factors could explain the observed discrepancies. For Fitbit’s step counts, the overestimation may be linked to its wrist-based placement. Compared to the waist-worn ActiGraph, a wrist-worn device reflects upper-limb activity more prominently, which can lead to false step recording [5]. This finding is consistent with other free-living studies that reported an overestimation of steps by Fitbit [20]. While some studies have reported underestimation, these were often based on short-duration protocols including specific exercises, suggesting that measurement tendencies vary by context. The significant overestimation of PAEE by Fitbit likely stems from its calculation method and the proportional bias we observed. Our finding that overestimation increased with higher activity levels aligns with previous research [10]. Given that our study protocol encouraged exercise, it is plausible that the relatively high levels of physical activity captured contributed to this pronounced overestimation. Conversely, the underestimation of MVPA by the Apple Watch in our free-living study presents an interesting contrast to some lab-based findings. In a treadmill study, the Apple Watch tended to recognize exercise time at relatively low thresholds, which could lead to overestimation during continuous exercise [21]. Our results suggest that under free-living conditions, where physical activity is often intermittent, the Apple Watch may fail to detect or accumulate sufficient minutes from these sporadic high-intensity episodes, resulting in an overall underestimation of MVPA.

It is also important to distinguish between the “absolute” and “relative” use of data from these consumer devices. For an individual user tracking their own daily activity, the primary value may lie in relative comparisons—for example, comparing today’s step count to yesterday’s. In this context, as long as the device is internally consistent, it can serve as a powerful motivational tool for behavior change, and its absolute agreement with a research-grade device is less of a concern. However, our findings serve as a crucial cautionary note for researchers intending to use these devices for studies where absolute values are paramount. The significant over- or underestimations and, notably, the proportional bias observed in metrics like Fitbit’s PAEE and Apple Watch’s MVPA, mean that data cannot be reliably compared across different devices or used as a direct substitute for criterion measures. This bias also implies that even for relative, within-individual tracking, the interpretation of changes could be skewed, as the degree of error may fluctuate with the intensity of activity.

The assessment of physical activity has become increasingly important in the study and management of a wide range of medical conditions and across various research fields [1]. However, our findings underscore the need for careful consideration when selecting devices for physical activity measurement. The choice of device should be guided by the specific output required, the level of precision needed, and the intended use of the data. Our results demonstrate that while consumer-grade devices perform well for step counting, they show varying levels of accuracy for MVPA and PAEE measurements. This variability suggests that researchers and clinicians should align their device selection with their primary outcomes of interest. In addition to accuracy, factors such as wearability, burden on participants, and potential compliance issues may play a crucial role in device selection. While research-grade devices like ActiGraph have established validity [2], they often require participants to wear an additional device specifically for the study, which can be less comfortable, especially when participants are already wearing their own personal devices in daily life. In contrast, consumer-grade wearables are gaining popularity among the general public [3], and leveraging existing device ownership could facilitate easier data collection through opt-in approaches. Researchers must balance usability with accuracy, considering the trade-offs between participant compliance and data precision. The selection of appropriate devices should be tailored to the specific research objectives, taking into account both the strengths and limitations of each option.

Future research in this field should address several key challenges. As new consumer-grade devices continually enter the market, there may be an ongoing need to compare their performance against reference devices. However, a more efficient approach would be for device manufacturers to conduct and disclose their own validation studies, calculation algorithms, and standardized exercise data. This transparency would eliminate the need for repeated comparative studies by independent researchers. While consumer-grade devices may be sufficient for general lifestyle monitoring, their integration into clinical trials and medical research requires a higher standard of validation. As wearable devices become increasingly prevalent in these fields, manufacturers may need to conduct more rigorous validation studies and provide greater transparency regarding their data processing algorithms. This shift towards openness and standardization could significantly enhance the utility and reliability of consumer-grade devices in research and clinical settings. As the field progresses, it will be crucial for researchers, clinicians, and device manufacturers to collaborate in establishing standards that meet both scientific rigor and practical applicability in various research and clinical contexts.

We also sought to contextualize our participants’ activity levels, measured during the COVID-19 pandemic, by comparing them with previously published data. We have summarized these comparisons for both MVPA and step counts in S3 Table. The table demonstrates that while MVPA values varied across studies, the activity levels of our participants were generally comparable to those reported in other relevant populations of healthy or working adults [6,8,19,22–25]. Therefore, we concluded that the pandemic did not appear to have substantially skewed the activity levels in our cohort relative to pre-pandemic findings.

### Limitations

This study has several limitations. First, it would have been useful to have all participants wear all four devices, allowing us to individually pool data across all participants and analyze per device, which would have eliminated most known and unseen confounders. However, the burden of wearing and maintaining four separate devices over a 3-week period would have been unfeasible for participants. Thus, in considering the study design, we needed to consider fairly balancing the need for robust data with the burden placed on participants. Second, a major limitation stems from comparing metrics derived from proprietary, “black box” algorithms, which forced methodological compromises for the Apple Watch (MVPA) and Fitbit (PAEE). The observed discrepancies are likely due to fundamental differences in these algorithms, such as Apple’s potential use of heart rate for MVPA or the compounding errors in Fitbit’s two-step PAEE calculation. Crucially, because manufacturers do not disclose their algorithms, any specific explanation for these differences remains speculative. This lack of transparency is a core methodological challenge, and thus, these findings must be interpreted with significant caution. Third, a critical limitation of this study is the small sample size. This was especially true for the Oura Ring (*n* = 5), for which the number of participants was substantially smaller than for the Apple Watch (*n* = 21) and Fitbit (*n* = 22). Furthermore, participants in the Oura Ring subgroup were not randomly assigned but were selected based on practical constraints (i.e., finger size and device availability), which introduces a potential for selection bias. Consequently, these factors prevent us from drawing definitive conclusions about the Oura Ring’s performance, and its findings should be considered preliminary and interpreted with significant caution. Fourth, the inclusion of only Japanese company employees (i.e., office workers) may limit the generalizability of the study to a broader population. Finally, the study took place during the COVID-19 pandemic and this may have influenced the activity levels of the participants, although based on comparisons with previous studies, the effect, if any, was likely to be small.

## Conclusions

The results of this study confirm that step counts from all three consumer-grade wearable devices—Apple Watch, Fitbit, and Oura Ring—were consistent with the research-grade ActiGraph. However, the ability of these devices to accurately measure MVPA and PAEE was limited and varied significantly by device. The Apple Watch substantially underestimated MVPA but overestimated PAEE. In contrast, the Fitbit showed a particularly large overestimation of PAEE, while its MVPA measurements were, on average, close to the ActiGraph’s. The Oura Ring tended to underestimate both MVPA and PAEE. Notably, our analysis revealed proportional bias for certain metrics, such as the Apple Watch’s MVPA and the Fitbit’s PAEE, where measurement errors increased at higher activity levels.

These findings underscore that while consumer-grade wearables offer promising opportunities for large-scale physical activity assessment, their use in research and clinical settings requires careful consideration and a clear understanding of their limitations. Researchers and clinicians must be aware of device-specific characteristics and potential biases, particularly when measuring metrics beyond simple step counts. The choice of a device must be guided by the specific metric of interest to ensure the validity of the collected data.

## Supporting information

S1 Table Summary of features of the four fitness tracking devices used in this study MVPA, moderate to vigorous physical activity; PAEE, physical activity energy expenditure. (PDF)

S2 Table Summary statistics for the ActiGraph GT9X activity data according to consumer-based wearable device subgroup Devices used were the ActiGraph GT9X, Apple Watch Series 6, Fitbit Sense and Oura Ring. Wear time was determined based on the ActiGraph data, as all devices were worn concurrently. Independent wear time data were not available for the consumer devices.
(PDF)

S3 Table Comparison of Physical Activity Levels with Previous Studies MVPA, moderate-to-vigorous physical activity; NHANES, National Health and Nutrition Examination Survey; NHNS, National Health and Nutrition Survey. (DOCX)
