# Supplementary material for: Comparison of consumer-grade wearable devices with a research-grade instrument for measuring physical activity in a free-living setting
Source: PLoS One. 2026 Feb 23;21(2):e0342543. doi: 10.1371/journal.pone.0342543 (PMC12928483; doi:10.1371/journal.pone.0342543)
Supplement: S1 Table — (PDF) [file pone.0342543.s001.pdf]

S1 Table. Summary of features of the four fitness tracking devices used in this study

	Device	File	Variable	Notes
STEP	AppleWatch	export.xml	StepCount	
	Fitbit	steps-[YYYY-MM-DD].json	value	
	Oura Ring	oura_[YYYY-MM-DD]T[HH-MM-SS].json	steps	
MVPA	AppleWatch	export.xml	AppleExerciseTime	This variable was used as a proxy for MVPA.
	Fitbit	moderately_active_minutes-[YYYY-MM-DD].json<br>very_active_minutes-[YYYY-MM-DD].json	value	Both were combined and analyzed as MVPA.
	Oura Ring	oura_[YYYY-MM-DD]T[HH-MM-SS].json	high,medium	
PEAA	AppleWatch	export.xml	ActiveEnergyBurned	This variable was used as a proxy for PAEE.
	Fitbit	calories-[YYYY-MM-DD].json	value	"'calories" refers to the total daily energy expenditure. PAEE was calculated by subtracting each participant's basal metabolic expenditure from their total daily energy expenditure
	Oura Ring	oura_[YYYY-MM-DD]T[HH-MM-SS].json	cal_active	

MVPA, moderate to vigorous physical activity; PAEE, physical activity energy expenditure
