# Supplementary material for: Comparison of consumer-grade wearable devices with a research-grade instrument for measuring physical activity in a free-living setting
Source: PLoS One. 2026 Feb 23;21(2):e0342543. doi: 10.1371/journal.pone.0342543 (PMC12928483; doi:10.1371/journal.pone.0342543)
Supplement: S2 Table — Wear time was determined based on the ActiGraph data, as all devices were worn concurrently. Independent wear time data were not available for the consumer devices. (PDF) [file pone.0342543.s002.pdf]

S2 Table. Summary statistics for the ActiGraph GT9X activity data according to consumer-based wearable device subgroup

		ActiGraph data			
**Subgroup**		**Mean (SD)**	**Median**	**Minimum**	**Maximum**
Apple Watch	MVPA (minutes/day)	52.39 (21.49)	46.6	20.9	97
	PAEE (kcal/day)	368.27 (116.03)	333.28	214.46	575.93
	Step count (steps/day)	7,610.77 (2608.99)	7,896.6	3,137.7	14,396.67
	Minutes worn/day	1,054.76 (115.07)	1,027.57	861.38	1,260
Fitbit	MVPA (minutes/day)	50.92 (24.87)	46.63	15.73	99.42
	PAEE (kcal/day)	367.34 (126.87)	342.51	146.41	649.36
	Step count (steps/day)	7,516.32 (2847.13)	7,045.36	3,237.11	14,396.67
	Minutes worn/day	1,085.95 (121.32)	1,111.69	883.94	1,301.38
Oura Ring	MVPA (minutes/day)	55.93 (26.18)	56.67	26.06	97
	PAEE (kcal/day)	380.45 (102.55)	447.38	248.29	461.83
	Step count (steps/day)	8,006.73 (2527.35)	8,226.69	4,646.41	11,533.65
	Minutes worn/day	1,051.57 (102.59)	1,046.06	912.76	1,185

Devices used were the ActiGraph GT9X, Apple Watch Series 6, Fitbit Sense and Oura Ring. Wear time was determined based on the ActiGraph data, as all devices were worn concurrently. Independent wear time data were not available for the consumer devices.

MVPA, moderate to vigorous physical activity; PAEE, physical activity energy expenditure; SD, standard deviation
