# Supplementary material for: Comparison of consumer-grade wearable devices with a research-grade instrument for measuring physical activity in a free-living setting
Source: PLoS One. 2026 Feb 23;21(2):e0342543. doi: 10.1371/journal.pone.0342543 (PMC12928483; doi:10.1371/journal.pone.0342543)
Supplement: S3 Table — (DOCX) [file pone.0342543.s003.docx]

**S3 Table. Comparison of Physical Activity Levels with Previous Studies.**

| Metric | Study (Reference) | Population | Device Used | Reported Outcome | Comparison with Present Study |
| --- | --- | --- | --- | --- | --- |
| MVPA | **Present Study** | **Japanese office workers** | **ActiGraph GT9X** | **53.3-62.6 min/day** | **-** |
|  | UK Adults [25] | Adults (29-64 yrs); predominantly sedentary/standing workers | Actiheart sensor | 83.4 (women), 124.0 (men) min/day | Higher |
|  | NHANES 2003-2004 [26] | USA adults | ActiGraph model 7164 | 15-24 (women), 26-43 (men) min/day | Lower |
|  | USA/Australia [6, 8, 27] | Healthy volunteers | ActiGraph GT3X | 37-87 min/day | Within Range |
| Steps | **Present Study** | **Japanese office workers** | **ActiGraph GT9X** | **7,513-7,814 steps/day** | **-** |
|  | Norway population [28] | Adults | ActiGraph GT1M | 8,314 steps/day | Comparable |
|  | Japan NHNS 2019 [29] | Japanese adults | Not specified in text | 7,864 (men), 6,685 (women) steps/day | Comparable |
|  | Australian study [27] | Healthy volunteers | ActiGraph GT3X | 7,511-8,497 steps/day | Comparable |
|  | Australian study [8] | Healthy volunteers | ActiGraph GT3X | 10,516 steps/day | Higher |

MVPA, moderate-to-vigorous physical activity; NHANES, National Health and Nutrition Examination Survey; NHNS, National Health and Nutrition Survey
